# Randomized prospective trial comparing mixed oil versus soybean oil lipid emulsion: A feasibility pilot study

**DOI:** 10.1016/j.intf.2026.100389

**Published:** 2026-08-07

**Authors:** Manpreet S. Mundi, Osman Mohamed Elfadil, Bradley R. Salonen, Darrell Schroeder, Sara L. Bonnes, Jennifer J. Carnell, Ryan T. Hurt

**Affiliations:** a Division of Endocrinology, Diabetes, Metabolism and Nutrition, Mayo Clinic, Rochester, MN, United States; b Division of General Internal Medicine, Mayo Clinic, Rochester, MN, United States; c Division of Clinical Trials and Biostatistics, Mayo Clinic, Rochester, MN, United States

**Keywords:** Home Parenteral Nutrition, Intestinal Failure, Intravenous Lipid emulsions, Fish Oil, IFALD

## Abstract

**Background:**

Mixed-oil (MO) intravenous lipid emulsions (ILEs) are increasingly used in adults receiving home parenteral nutrition (HPN), but prospective data from United States HPN programs remain limited. This feasibility pilot trial randomized adults newly initiated on HPN to MO versus soybean-oil (SO) ILE. The prespecified primary endpoint was change in total bilirubin over 12 weeks; secondary endpoints included feasibility, tolerance/safety, liver enzymes, C-reactive protein (CRP), and fatty acid profile.

**Methods:**

This was a pragmatic, single-center, blinded, randomized, prospective trial of adults newly initiated on HPN and expected to require HPN for more than 3 months. Participants were randomized to SO ILE or MO ILE. The standard HPN follow-up protocol was used to assess tolerance, complications, laboratory outcomes, and fatty acid profiles at baseline and through 12 weeks. Analyses included randomized participants with available data at each time point.

**Results:**

Twenty-two participants were randomized (SO ILE n = 11; MO ILE n = 11). Groups were similar in baseline demographic and PN macronutrient characteristics, and all participants had normal baseline liver enzymes and bilirubin. Total bilirubin, the primary endpoint, did not differ significantly between groups at weeks 4, 8, or 12 (week 12 median change 0 [IQR −0.1, 0.1] in SO ILE vs 0 [0, 0.1] in MO ILE; p = 0.96). No significant between-group differences were observed in liver enzymes or CRP. MO ILE was associated with significant increases in EPA and DHA by week 4 that were maintained through week 12, whereas SO ILE was not.

**Conclusion:**

In this feasibility pilot study, MO ILE was tolerated in United States adults newly initiated on HPN and produced the expected increase in EPA and DHA, with potential benefit in reduction of inflammation in the setting of inflammatory insult. Further multi-center trials are needed to confirm these findings and explore long-term outcomes.

## Introduction

Intestinal failure (IF) is defined as the reduction of gastrointestinal function below the minimum necessary for the absorption of macronutrients, water, and/or electrolytes, requiring intravenous supplementation [1]. Chronic intestinal failure (CIF) with persistence of IF for months or years requiring home parenteral nutrition (HPN) to serve a supportive and life-sustaining role is estimated to occur in approximately 25,000 Americans (75 per million) [2], [3]. Since the development of a stable and safe intravenous 100% soybean oil (SO) lipid emulsion (ILE) in the 1960s, ILEs have served as an integral part of HPN [4], [5]. ILEs serve as a key source of essential fatty acids as well as non-protein calories, allowing for a reduction in the dependence on dextrose for non-protein calories, which can be associated with hyperglycemia, essential fatty acid deficiency (EFAD), and liver abnormalities [6], [7].

Despite these benefits, long-term use of SO ILE can be associated with complications, including infection, inflammation, hypertriglyceridemia, and IF-associated liver disease (IFALD) [4]. Although IFALD remains a diagnosis of exclusion, as many as 30–60% of children and 15–40% of adults with CIF can develop IFALD with presentations including hepatic steatosis, cholestasis, cholelithiasis, and hepatic fibrosis [6], [8]. The etiology of IFALD is multi-factorial with some studies demonstrating a correlation with bowel length of less than 50 cm and ILE intake of ≥ 1 g/kg/day [9]. Elevated levels of phytosterols may also be contributing as higher levels correlate with the severity of IFALD [10], [11], [12]. Additionally, the high omega−6 polyunsaturated fatty acid (PUFA) content of SO ILE can be pro-inflammatory as linoleic acid is converted to arachidonic acid and further metabolized to generate proinflammatory eicosanoids [13]. Previous studies have also noted a theoretical increased risk of infection and poor clinical outcomes through a direct effect on membrane fluidity and immune suppression [13].

These concerns have led to changes in the provision of ILEs, with recommendations to limit SO ILE to ≤ 1 g/kg/day and many clinical practices utilizing SO ILE three days or fewer per week [1], [14]. Development of alternative ILEs focused on strategies of decreasing reliance on SO and utilizing lipid sources with theoretical benefits, such as medium chain triglycerides (MCT), olive oil (OO), and fish oil (FO), has also taken place over the last few decades [15]. The latest ILE to be approved has used a mixed oil (MO) approach combining SO, MCT, OO, and FO in order to minimize theoretical risk while maximizing clinical benefit. Use of MO ILE in several short-term trials has demonstrated good safety and tolerance data, with some studies showing lower liver function studies compared to SO ILE [16], [17], [18], [19], [20], [21]. Additionally, case series have demonstrated that in patients with intolerance to SO ILE, transitioning to MO ILE led to an increase in non-protein calories from ILE and a reduction in dextrose energy, while liver studies were either maintained or improved [22], [23].

Given limited prospective data regarding MO ILE from United States adult HPN programs, the current pilot randomized controlled trial was designed primarily to compare the effect of MO versus SO ILE on total bilirubin over 12 weeks. Secondary aims were to assess recruitment and retention feasibility, tolerance and safety, liver enzymes, CRP, and fatty acid profiles. Because enrolled participants were newly initiated on HPN and excluded for clinically apparent liver dysfunction, the study should be interpreted as a feasibility and early-safety study rather than a definitive trial of IFALD prevention or treatment.

## Methods

### Design

**Fig. 1:**
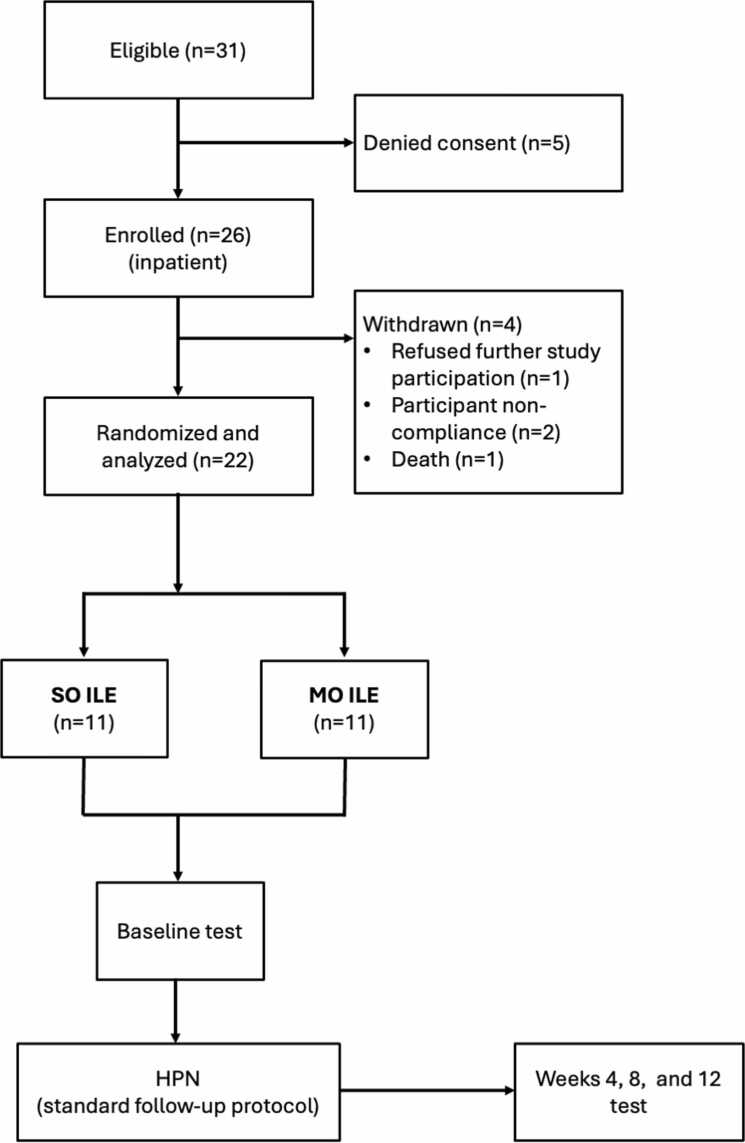
Study process flow chart.

This was a single-center, blinded, prospective trial randomizing participants initiating HPN to SO ILE or MO ILE; screening, enrollment, randomization, and follow-up are summarized in Fig. 1. Only the team pharmacist, acting as the data safety monitor, knew the ILE assignment. Randomization was not stratified by intestinal failure etiology or anatomical type because of the pilot sample size. The study was approved by Mayo Clinic Institutional Review Board (IRB # 17–001403) and was registered on ClinicalTrials.gov (**NCT03773237**). The SO ILE utilized was Intralipid (Baxter/Fresenius Kabi USA) composed of triglycerides (100% SO), 1.2 g egg yolk phospholipids, and 2.25 g glycerin. The MO ILE utilized was SMOFlipid (Fresenius Kabi USA, Lake Zurich, Illinois), composed of triglycerides (30% SO, 30% MCT, 25% OO, and 15% FO), 1.2 g egg phospholipids, and 2.5 g glycerin. The study was pragmatically designed and followed the standard HPN protocol [24], with baseline bloodwork collection followed by transition to study-defined ILE (Supplemental Table 1). Participants were followed within 1 week of discharge and had monthly check-ins to assess HPN tolerance and monitor for complications; additional follow-up was performed as clinically indicated.

### Participants

All adult patients (age 18–75 years) newly initiated on HPN for intestinal failure and expected by the treating HPN team at the time of discharge to require HPN for more than 3 months were offered participation if they did not meet exclusion criteria (Fig. 1). Chronic intestinal failure was defined in accordance with ESPEN as persistent reduction of gut function below the minimum necessary for absorption of macronutrients and/or water and electrolytes such that intravenous supplementation is required to maintain health in a metabolically stable patient^1^. Because participants were enrolled at HPN initiation, expected HPN duration greater than 3 months was determined prospectively from the underlying diagnosis, anticipated intestinal rehabilitation course, and treating team assessment. Participants were excluded if they were age < 18 years, pregnant or lactating, had active malignancy, expected duration of HPN less than 3 months, proven alcohol addiction/dependence with active use in the prior 12 months, known hypersensitivity to fish, egg, soybean, or peanut protein, active infection, clinically significant liver dysfunction, or receipt of MO ILE for more than 2 weeks in the prior 12 months. For screening, clinically significant liver dysfunction was operationalized as liver function studies > =2 times the upper limit of normal, radiologic or histologic evidence of active liver pathology, or investigator-determined clinically significant liver disease. This safety exclusion was not intended to diagnose IFALD; IFALD was considered using the ESPEN-endorsed concept of abnormal liver function tests and/or radiologic or histologic liver abnormalities in a patient with intestinal failure after exclusion of other primary liver pathology, hepatotoxic exposures, or biliary obstruction^8^.

### Objectives

***Primary aim***: The prespecified primary objective was to compare the change in total bilirubin between MO ILE and SO ILE over 12 weeks. ***Secondary aims***: Secondary objectives were to evaluate recruitment and retention feasibility, tolerance and safety of the assigned ILE, and changes in fatty acids, alkaline phosphatase (ALP), alanine aminotransferase (ALT), aspartate aminotransferase (AST), gamma-glutamyl transferase (GGT), and CRP. Given the pilot design and small sample size, all efficacy analyses were considered exploratory.

### Statistical analysis

Continuous variables are summarized as mean + /- standard deviation (SD) when approximately normally distributed and as median (interquartile range [IQR]) otherwise. Categorical variables are summarized as n (%). Laboratory data are summarized separately for each treatment group using median (IQR: 25th, 75th). The change from baseline was calculated for each subject at weeks 4, 8, and 12. Analyses followed a modified intention-to-treat approach: all randomized participants with available data at each time point were included, and missing values were not imputed. For each treatment group, the change from baseline was compared with zero using the signed-rank test; changes from baseline were compared between treatment groups using the rank-sum test. In all cases, a two-tailed p < 0.05 was considered statistically significant.

## Results

### Baseline patient characteristics

Thirty-one patients met eligibility criteria and were invited to participate (Fig. 1). Five patients declined to participate and did not provide consent, whereas four withdrew after enrollment for reasons including a participant's request for withdrawal and noncompliance with study visits. One participant died due to their underlying disease. A total of 22 participants completed the study and were randomized to SO ILE (n = 11) or MO ILE (n = 11). Two participants in each group discontinued HPN before the 12-week assessment despite a prospective expectation at enrollment that HPN would be required for more than 3 months due to improved oral/enteral intake. These participants were included in analyses through their last available laboratory assessment. At baseline, the SO and MO groups were comparable with respect to age, sex distribution, BMI, baseline PN macronutrient prescription, liver studies, CRP, and fatty acid profile (Table 1). Intestinal failure etiology, colon continuity, and HPN indication, are also summarized in Table 1. Notably, all participants in this study were totally fasting at enrollment and 9 patients in each group (81.8%) continued to be totally fasting during the study.

**Table 1:** Baseline demographics, clinical/laboratory and PN t characteristics*.

	SO ILE	MO ILE	
Characteristic	(N = 11)	(N = 11)	p-value
Age, years	57.5 ± 16.5	57.9 ± 14.7	0.96
Sex			1.00
Female	8 (73%)	7 (64%)	
Male	3 (27%)	4 (36%)	
BMI, kg/m^2^	20.1 ± 8.1	24.4 ± 9.4	0.27
Primary pathophysiological mechanism/disease process, n (%)			
• Mechanical obstruction	3 (27.3)	2 (18.2)	
• Enterocutaneous fistula	2 (18.2)	2 (18.2)	
• Mucosal disease	3 (27.3)	4 (36.6)	
• SBS	1 (9)	1 (9)	
• Dysmotility	2 (18.2)	1 (9)	
• Bariatric surgery (complications)	-	1 (9)	
Indication for HPN initiation, n (%)			
• SBS	5 (45.4)	5 (45.4)	
• Intestinal failure/Malnutrition	3 (27.3)	2 (18.2)	
• Severe malabsorptive state	3 (27.3)	4 (36.4)	
Colon in continuity, n (%)	8 (72.7)	9 (81.8)	0.61
Amino Acids (g/kg/d)	1.4 ± 0.3	1.4 ± 0.2	0.54
Dextrose (g/kg/d)	4.8 ± 1.4	3.9 ± 1.3	0.14
Lipid (g/kg/d)	0.9 ± 0.3	0.8 ± 0.3	0.55
Total calories (kcal/kg/d)	30.1 ± 8.5	26.4 ± 8.4	0.31
Alk Phos	84.0 (63.0, 107.0)	101.0 (81.0, 148.0)	0.22
ALT	20.0 (11.0, 35.0)	17.0 (11.0, 43.0)	0.90
AST	21.0 (17.0, 33.0)	19.0 (13.0, 45.0)	0.90
Total Bilirubin	0.2 (0.2, 0.4)	0.2 (0.2, 0.4)	0.63
GGT	20.0 (17.0, 31.0)	31.0 (22.0, 138.0)	0.13
CRP	5.7 (3.0, 10.9)	9.4 (3.8, 29.5)	0.30
DHA	166.0 (113.0, 228.0)	169.0 (151.0, 194.0)	0.82
EPA	31.0 (22.0, 84.0)	50.0 (28.0, 104.0)	0.37
Linoleic Acid	2620.0 (1913.0, 3663.0)	2568.0 (2037.0, 3150.0)	0.58
α-Linolenic Acid	82.0 (54.0, 131.0)	58.0 (48.0, 103.0)	0.25
Arachidonic Acid	760.0 (609.0, 996.0)	813.0 (688.0, 998.0)	0.31
Mead Acid	20.0 (16.0, 25.0)	22.0 (19.0, 24.0)	0.72

* Age and BMI are summarized using mean ± SD and compared between groups using the two-sample *t*-test. Sex is summarized using n (%) and compared between groups using Fisher’s exact test. *Data are summarized using median (25th, 75th) and compared between groups using the rank sum test.

### HPN regimen

The SO and MO groups received similar PN regimens with respect to average number of PN and lipid infusion days per week, total volume, and nutrient content, including daily amino acids and lipid dose (Table 1). There was no significant difference between groups in PN calories delivered as kcal/day, weekly average kcal/day, or weekly average kcal/kg/day. No overall change in formula regimen in weeks 4, 8, and 12 was observed between the SO and MO groups (Supplementary Table 2). As HPN-related complications, including catheter-related bloodstream infections or thrombosis, were prospectively captured during follow-up, 2 (18.2%) patients in each group developed and treated for an episode of CRBSI during the study. No catheter related thrombosis were noted in all study participants and across both study groups.

### Biochemical findings

#### Baseline

At baseline, no significant between-group differences were observed in key laboratory variables used to assess the assigned ILEs (Table 1). All participants (n = 22) had normal liver enzymes and total bilirubin at baseline. Median CRP was 5.7 (IQR 3.0, 10.9) in the SO ILE group and 9.4 (3.8, 29.5) in the MO ILE group (p = 0.30). No significant baseline differences were observed in fatty acid profiles between groups.

##### CRP

**Fig. 2:**
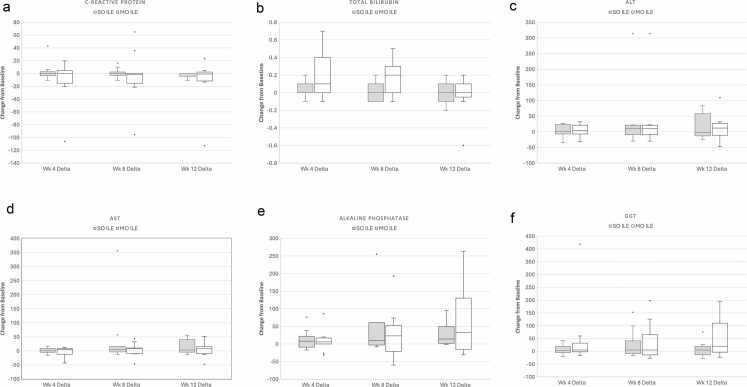
Change in liver studies from baseline.

CRP did not differ significantly between groups over follow-up (Table 2 **and** Fig. 2**a**). Median CRP change from baseline in the SO ILE group was 0 (−3.5, 2.7) at week 4, 0 (−3.1, 2.4) at week 8, and 0 (−3.2, 0) at week 12. Median CRP change in the MO ILE group was 0 (−15.3, 4.7) at week 4, −1.5 (−15.3, 0.4) at week 8, and −0.8 (−9.9, 0) at week 12. Between-group p values were 0.49, 0.19, and 0.96 at weeks 4, 8, and 12, respectively.

**Table 2:** Lab value changes from baseline at 4, 8, and 12 weeks.

	SO ILE			MO ILE		
Characteristic	N	Median (25th, 75th)		N	Median (25th, 75th)	p-value*
Alk Phos						
Week 4 delta	11	8.0 (−9.0, 22.0)		11	6.0 (0, 17.0)	1.00
Week 8 delta	11	10.0 (−3.0, 61.0)**		11	23.0 (−21.0, 53.0)	1.00
Week 12 delta	9	14.0 (4.0, 25.0)**		9	33.0 (−15.0, 74.0)	0.79
ALT						
Week 4 delta	11	2.0 (−7.0, 23.0)		11	4.0 (−7.0, 21.0)	0.87
Week 8 delta	11	10.0 (−9.0, 21.0)		11	3.0 (−1.0, 27.0)	0.79
Week 12 delta	9	−2.0 (−7.0, 32.0)		9	12.0 (−3.0, 21.0)	0.86
AST						
Week 4 delta	11	0 (−5.0, 9.0)		11	5.0 (−12.0, 9.0)	0.87
Week 8 delta	11	4.0 (−4.0, 16.0)		11	8.0 (−10.0, 10.0)	0.49
Week 12 delta	9	3.0 (−4.0, 26.0)		9	8.0 (−6.0, 13.0)	0.79
Total Bilirubin						
Week 4 delta	11	0 (0, 0.1)		11	0.1 (0, 0.4)**	0.18
Week 8 delta	11	0 (−0.1, 0.1)		11	0.2 (0, 0.3)**	0.09
Week 12 delta	9	0 (−0.1, 0.1)		9	0 (0, 0.1)	0.96
GGT						
Week 4 delta	11	4.0 (−5.0, 19.0)		11	5.0 (−2.0, 32.0)	0.53
Week 8 delta	11	5.0 (−9.0, 41.0)		11	5.0 (−14.0, 65.0)	0.92
Week 12 delta	9	5.0 (−13.0, 12.0)		9	20.0 (2.0, 67.0)	0.27
CRP						
Week 4 delta	11	0 (−3.5, 2.7)		11	0 (−15.3, 4.7)	0.49
Week 8 delta	11	0 (−3.1, 2.4)		11	−1.5 (−15.3, 0.4)	0.19
Week 12 delta	9	0 (−3.2, 0)		9	−0.8 (−9.9, 0)	0.96
DHA						
Week 4 delta	11	4.0 (−21.0, 70.0)		10	195.5 (150.0, 344.0)**	**0.006**
Week 8 delta	10	−20.5 (−43.0, 17.0)		11	231.0 (27.0, 284.0)**	**0.007**
Week 12 delta	9	−2.0 (−11.0, 41.0)		8	218.5 (54.5, 374.0)**	**0.006**
EPA						
Week 4 delta	11	−3.0 (−12.0, 15.0)		10	143.0 (69.0, 218.0)**	**< .001**
Week 8 delta	10	5.0 (−39.0, 14.0)		11	129.0 (78.0, 226.0)**	**0.001**
Week 12 delta	9	2.0 (−22.0, 6.0)		8	197.5 (96.0, 341.5)**	**0.005**
Linoleic Acid						
Week 4 delta	11	−108.0 (−408.0, 294.0)		10	5.5 (−331.0, 443.0)	0.48
Week 8 delta	10	−42.5 (−598.0, 587.0)		11	−65.0 (−610.0, 611.0)	0.94
Week 12 delta	9	115.0 (−661.0, 263.0)		8	247.0 (−3.0, 1134.5)	0.29
A-Linolenic Acid						
Week 4 delta	11	−19.0 (−41.0, 1.0)		10	−2.5 (−29.0, 12.0)	0.44
Week 8 delta	10	−8.0 (−77.0, 27.0)		11	−4.0 (−10.0, 47.0)	0.36
Week 12 delta	9	−37.0 (−66.0, −6.0)		8	5.5 (−18.0, 51.5)	**0.030**
Arachidonic Acid						
Week 4 delta	11	107.0 (−26.0, 163.0)		10	197.5 (−84.0, 545.0)	0.29
Week 8 delta	10	−13.0 (−107.0, 278.0)		11	147.0 (−142.0, 355.0)	0.62
Week 12 delta	9	111.0 (37.0, 184.0)		8	395.0 (238.5, 476.0)**	0.08
Mead Acid						
Week 4 delta	11	1.0 (−2.0, 6.0)		10	3.0 (−4.0, 14.0)	0.92
Week 8 delta	10	−3.0 (−6.0, 4.0)		11	6.0 (−4.0, 10.0)	0.13
Week 12 delta	9	−1.0 (−5.0, 7.0)		8	11.5 (4.0, 21.5)**	0.12

*P-value from rank sum test comparing the given change from baseline between groups.

**p < 0.05 from signed rank test comparing the given change from baseline to zero.

### Liver studies

Total bilirubin, the primary endpoint, did not differ significantly between groups at any follow-up time point (Table 2 **and** Fig. 2**b**). Median change in total bilirubin at weeks 4, 8, and 12 was 0 (0, 0.1), 0 (−0.1, 0.1), and 0 (−0.1, 0.1) in the SO ILE group versus 0.1 (0, 0.4), 0.2 (0, 0.3), and 0 (0, 0.1) in the MO ILE group, respectively; between-group p values were 0.18, 0.09, and 0.96. No significant between-group differences were observed for ALP, ALT, AST, or GGT change from baseline at week 12 (p = 0.79, 0.86, 0.79, and 0.27, respectively; **(**Fig. 2**c-f**).

### Fatty acids

**Fig. 3:**
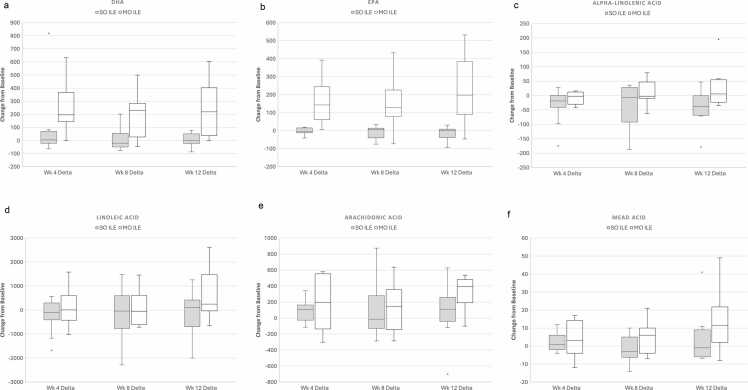
Change in fatty acid levels from baseline.

After randomization, MO ILE was associated with significant increases in omega−3 PUFAs, including DHA and EPA (Table 2 **and** Fig. 3**a-b**). Median DHA change in the MO ILE group was 195.5 (150.0, 344.0) at week 4, 231.0 (27.0, 284.0) at week 8, and 218.5 (54.5, 374.0) at week 12; corresponding between-group p values were 0.006, 0.007, and 0.006. Median EPA change in the MO ILE group was 143.0 (69.0, 218.0), 129.0 (78.0, 226.0), and 197.5 (96.0, 341.5) at weeks 4, 8, and 12; corresponding between-group p values were < 0.001, 0.001, and 0.005. Alpha-linolenic acid decreased in the SO ILE group and was significantly different from MO ILE at week 12 (p = 0.030; Fig. 3**c**). Linoleic acid, arachidonic acid, and mead acid changes did not differ significantly between groups (Table 2 **and** Fig. 3**d-f**).

## Discussion

This pilot randomized trial compared MO ILE with SO ILE in adults newly initiated on HPN at a large tertiary referral center in the United States. The prespecified primary endpoint, change in total bilirubin over 12 weeks, did not differ significantly between groups. The most consistent biochemical finding was the expected increase in EPA and DHA in the MO ILE group. MO ILE was tolerated during the study period, but the trial should be interpreted primarily as a feasibility and early safety pilot rather than evidence of clinical superiority for liver or inflammatory outcomes. Recruitment was difficult, partially because of the COVID−19 pandemic and partially because some patients declined enrollment due to the possibility of randomization to SO ILE.

Prospective randomized data in adult HPN cohorts remain limited compared with inpatient and critically ill populations. Previous adult HPN studies comparing MO-containing ILEs with SO or other lipid emulsions have been conducted outside the United States and have varied in design, lipid dose, and follow-up duration [21], [25], [26], [27]. The current study adds a pragmatic United States experience in adults newly initiated on HPN and provides feasibility data for trial design in this setting. Initially, Klek et al. carried out a multi-center trial involving IF centers in Europe that randomized 73 participants to either SO ILE or MO ILE for 4 weeks with results published in 2013 [21]. Osowska et al. randomized 32 HPN participants recruited from two Polish Intestinal Failure Centers to transition from SO ILE to either MO ILE or SO/OO ILE for 60 days [25]. In 2021, Klek et al. published the results of a RCT that randomized 67 participants receiving HPN at a single Polish IF center to either MCT/SO, OO/SO, or MO ILE with 5 years of follow-up, the longest follow-up study to date [26]. The latest study published by Clermont-Dejean et al. utilized a crossover design where 65 participants from an IF center in Toronto, Canada, were randomized to receive either SO ILE or MO ILE for 6 months, followed by a 1-month washout period and then crossover to the other ILE [27].

Given the association of ILE use and the development of IFALD, all studies closely monitored liver function tests. In the current study, we noted that all participants had normal liver enzymes as well as total bilirubin levels at baseline. Although there was no significant difference in liver enzymes between the two groups in follow-up, we did notice a gradual increase in liver enzymes in both groups until 8 weeks, followed by a plateau. Similar findings regarding liver studies were noted in the other MO ILE trials. Osowaska et al. reported that most liver studies remained stable for 60 days after transition to either MO ILE or SO/OO ILE from SO ILE [25]. In the SO/OO ILE group, they noted that GGT in the SO/OO group was initially elevated at 69 (46.5–141.0) U/L and then improved to 49.0 (27.5, 109.0) U/L. In a case-control study of 17 patients with intolerance to SO ILE, transitioning to MO ILE increased ILE-calorie provision and was associated with improvements in AST, ALT, and total bilirubin [23]. Similarly, Klek et al. in the long-term HPN study noted that LFTs normalized over time with only significant finding being a lower mean bilirubin level in the MO ILE group [26]. The short-term RCT by Klek et al. demonstrated that mean concentrations of ALT, AST, and total bilirubin were significantly lower with the MO ILE group after only 28 days [21]. It is important to note that this study provided ILE doses of 1.3 g/kg/day on average, which was significantly higher than the ILE dose provided in other studies, including ours, which ranged between 0.6 and 0.9 g/kg/day.

The clearest secondary endpoint finding was the increase in omega−3 fatty acid levels in the MO ILE group. DHA and EPA increased by week 4 and remained elevated through week 12, whereas levels in the SO ILE group remained stable or decreased. This pattern is consistent with the fatty acid composition of MO ILE and with prior HPN studies reporting increased EPA and DHA after exposure to fish-oil-containing lipid emulsions [21], [25], [27]. The α-linolenic level also increased in the MO ILE from baseline, while decreasing in the SO ILE group over 12 weeks. Additionally, linoleic acid levels rose in both groups, although there was significant variability in the change from baseline. Given higher OO content, the mead acid level also increased in the MO ILE group and remained stable in the SO ILE group. This, combined with an increase in Arachidonic acid levels in the MO ILE group, perhaps contributed to the lack of clinical or biochemical signs of EFAD.

Osowska et al., due to its unique design, noted that transferring from SO ILE to MO ILE led to a decrease in linoleic acid, α-linolenic acid, and mead acid while resulting in significant increases in DHA and EPA [25]. Interestingly, transfer from SO ILE to OO/SO ILE resulted in a decline in linoleic acid, arachidonic acid, mead acid, as well as both EPA and DHA. Both Klek et al. and Clermont-Dejean et al. noted that there was a decrease in linoleic acid, and arachidonic acid, as well as an increase in EPA and DHA levels with MO ILE [21], [27]. The SO ILE group showed increases in linoleic acid, α-linolenic acid, and arachidonic acid levels along with decreases in EPA and DHA levels, reflecting the composition of these ILE. Similar to our findings, no study reported the development of biochemical or clinical evidence of EFAD.

The increase in EPA and DHA with MO ILE is biologically plausible and may be relevant to future mechanistic studies, as omega−3 fatty acids can serve as precursors for less inflammatory lipid mediators and specialized pro-resolving mediators [13], [28]. However, any anti-inflammatory implications of MO ILE in this cohort should be considered hypothesis-generating only due to lack of power to detect significant anti-inflammatory implications.

The high omega−6 polyunsaturated fatty acid (PUFA) content of SO ILE can, theoretically, be associated with poor clinical outcomes including increased inflammation. Metabolites of linoleic acid can generate proinflammatory eicosanoids including 2-series prostaglandins and thromboxanes and 4-series leukotrienes [13]. Higher omega−3 PUFA content of FO tends to be less inflammatory, generating 3-series prostaglandins and thromboxanes and 5-series leukotrienes. EPA and DHA also give rise to specialized pro-resolving molecules (SPMs), which not only dampen acute leukocyte responses but also facilitate the active resolution of the inflammatory response. SPMs include resolvins, protectins, and maresins with E- and D-series resolvins produced from EPA and DHA respectively, while protectins and maresins are produced from DHA [28]. Their inflammatory benefit is noted through a number of mechanisms including reduced cytokine production, decreased neutrophil infiltration, and enhancement of phagocytic and clearance function of macrophages [13].

The impact of FO-augmented ILE on inflammation was demonstrated quite well in several clinical trials, including a crossover-controlled study of 12 patients on long-term HPN [29]. All patients received MO ILE for 12 weeks, which was augmented with 100% FO ILE for 4 weeks. After those 4 weeks, they had each patient transition to another MO ILE with 10% FO content for 6 weeks, followed by 6 weeks on SO/OO ILE. Each of the subsequent cycles also were augmented with 10 g of 100% FO ILE. The addition of 100% FO ILE led to a reduction in the production of interleukin (IL)−1, IL-6, and tumor necrosis factor (TNF)-α in response to lipopolysaccharide (LPS) stimulation. Pastor-Clerigues et al. studied the impact of 100% FO ILE on LPS-induced inflammatory cytokines in monocytes from healthy participants and reported a reduction in the release of IL-6, TNF-α, and IL-8 [30]. Additional clinical trials have shown that this reduction in cytokines correlates with an increase in SPMs in response to EPA and DHA supplementation [31], [32]. In each of these studies, FO prevented the rise in cytokines in response to an inflammatory insult, such as that mediated by LPS stimulation. This could explain why CRP remained stable in our study over 12 weeks, as our cohort did not experience inflammatory stress during study follow-up. The MO ILE group had a slightly higher CRP level at baseline, which normalized over time.

### Limitations

This study has several important limitations. First, although total bilirubin was the prespecified primary endpoint, the study population had normal baseline liver tests, received relatively low lipid doses, and was followed for only 12 weeks. The trial was therefore not powered or optimally designed to detect clinically meaningful IFALD prevention or treatment effects. Second, the sample size was small, and recruitment was difficult. Recruitment began in 2018 and slowed substantially during the COVID−19 pandemic. As the HPN program recovered, increasing familiarity with MO ILE led some patients to decline a trial in which they might be randomized to SO ILE, and clinicians also raised concerns about continued randomization in light of emerging data favoring fish-oil-containing ILEs [33]. Third, participants were newly initiated on HPN and were enrolled based on expected HPN duration greater than 3 months; four participants discontinued HPN before 12 weeks, underscoring the challenge of prospectively identifying a stable chronic intestinal failure cohort at HPN initiation. Fourth, the pilot sample limits adjustment for intestinal failure etiology, residual bowel anatomy, colon continuity, oral/enteral intake, and infectious complications, all of which can influence liver-related outcomes. These factors support the need for larger multicenter studies with longer follow-up, enriched liver-risk cohorts, and standardized chronic intestinal failure and IFALD characterization.

## Conclusions

In conclusion, this blinded prospective randomized pilot trial found that MO ILE was tolerated in United States adults newly initiated on HPN and produced the expected increase in EPA and DHA levels. The study did not show significant between-group differences in the prespecified primary endpoint of total bilirubin or in other liver enzymes or CRP over 12 weeks. Because participants had normal baseline liver tests, received relatively low lipid doses, and were followed for a short interval, the findings should be interpreted as feasibility and early safety data rather than evidence of clinical superiority for IFALD or inflammation outcomes. Larger multicenter trials with longer follow-up and detailed intestinal failure characterization are needed to evaluate clinical outcomes.

## CRediT authorship contribution statement

**Manpreet S. Mundi:** Writing – review & editing, Writing – original draft, Supervision, Resources, Project administration, Methodology, Investigation, Funding acquisition, Data curation, Conceptualization. **Ryan T. Hurt:** Writing – review & editing, Validation, Supervision, Conceptualization. **Jennifer J. Carnell:** Writing – review & editing, Data curation. **Sara L. Bonnes:** Writing – review & editing, Data curation. **Darrell Schroeder:** Writing – review & editing, Formal analysis, Data curation. **Bradley R. Salonen:** Writing – review & editing, Data curation. **Osman Mohamed Elfadil:** Writing – review & editing, Writing – original draft, Project administration, Investigation, Formal analysis, Data curation.

## Ethical approval

The study was approved by Mayo Clinic Institutional Review Board (IRB # 17–001403) and was registered on ClinicalTrials.gov (**NCT03773237**).

## Financial disclosures

MSM – has research grants from Nestle, Fresenius Kabi, VectivBio, Rockfield, and Realfood Blends. He is on advisory board of NorthSea, NutriShare, and Otsuka.

RTH – is a consultant for Nestle and has research grant from Zealand.

OME – has no disclosures pertinent to this manuscript.

BRS – has no disclosures pertinent to this manuscript.

DS – has no disclosures pertinent to this manuscript.

SLB – has no disclosures pertinent to this manuscript.

JJC – has no disclosures pertinent to this manuscript.

## Funding

This study was funded by Fresenius Kabi through Investigator Initiated Grant.

## Footnotes

Appendix A Supplementary data associated with this article can be found in the online version at doi:10.1016/j.intf.2026.100389.
