# Supplementary material for: Randomized prospective trial comparing mixed oil versus soybean oil lipid emulsion: A feasibility pilot study
Source: Intest Fail. 2026 Aug 7;12:100389. doi: 10.1016/j.intf.2026.100389 (PMC13476644; doi:10.1016/j.intf.2026.100389)
Supplement: Supplementary file 1 [file mmc1.docx]

Supplementary Table 1: Laboratory Assessment Schedule

| Baseline | Week 1 | Week 2 | Week 3 | Week 4 | Week 6 | Week 8 | Week 10 | Week 12 |
| --- | --- | --- | --- | --- | --- | --- | --- | --- |
| Sodium | Sodium | Sodium | Sodium | Sodium | Sodium | Sodium | Sodium | Sodium |
| Potassium | Potassium | Potassium | Potassium | Potassium | Potassium | Potassium | Potassium | Potassium |
| Creatinine | Creatinine | Creatinine | Creatinine | Creatinine | Creatinine | Creatinine | Creatinine | Creatinine |
| BUN | BUN | BUN | BUN | BUN | BUN | BUN | BUN | BUN |
| Bicarbonate | Bicarbonate | Bicarbonate | Bicarbonate | Bicarbonate | Bicarbonate | Bicarbonate | Bicarbonate | Bicarbonate |
| Chloride | Chloride | Chloride | Chloride | Chloride | Chloride | Chloride | Chloride | Chloride |
| AST | Phosphorus | AST | Phosphorus | AST | AST | AST | AST | AST |
| ALT | Calcium | ALT | Calcium | ALT | ALT | ALT | ALT | ALT |
| Total Bilirubin | Magnesium | Total Bilirubin | Magnesium | Total Bilirubin | Total Bilirubin | Total Bilirubin | Total Bilirubin | Total Bilirubin |
| Alk Phos |  | Alk Phos |  | Alk Phos | Alk Phos | Alk Phos | Alk Phos | Alk Phos |
| Phosphorus |  | Phosphorus |  | Phosphorus | Phosphorus | Phosphorus | Phosphorus | Phosphorus |
| Calcium |  | Calcium |  | Calcium | Calcium | Calcium | Calcium | Calcium |
| Glucose |  | Glucose |  | Glucose | Glucose | Glucose | Glucose | Glucose |
| Total Protein |  | Total Protein |  | Total Protein | Total Protein | Total Protein | Total Protein | Total Protein |
| Albumin |  | Albumin |  | Albumin | Albumin | Albumin | Albumin | Albumin |
| CBC w/Diff |  | CBC w/Diff |  | CBC w/Diff | CBC w/Diff | CBC w/Diff | CBC w/Diff | CBC w/Diff |
| Magnesium |  | Magnesium |  | Magnesium | Magnesium | Magnesium | Magnesium | Magnesium |
| TAG |  | TAG |  | TAG | TAG | TAG | TAG | TAG |
| CRP |  | CRP |  | CRP | CRP | CRP | CRP | CRP |
| Insulin |  |  |  |  |  |  |  | ***STR** |
| c-peptide |  |  |  |  |  |  |  | *****Copper*** |
| Triene/Tetrane ratio |  |  |  |  |  |  |  | *****Zinc*** |
| phytosterol |  |  |  |  |  |  |  | *****Selenium*** |
|  |  |  |  |  |  |  |  | *****Manganese*** |
|  |  |  |  |  |  |  |  | Vitamin A |
|  |  |  |  |  |  |  |  | Vitamin E |
|  |  |  |  |  |  |  |  | 25-HydroxyD2,D3 |
|  |  |  |  |  |  |  |  | insulin |
|  |  |  |  |  |  |  |  | C-peptide |
|  |  |  |  |  |  |  |  | Triene/Tetrane ratio |
|  |  |  |  |  |  |  |  | phytosterol |

Supplementary Table 2: Change in parenteral nutrition regimen over 12 weeks

| Characteristic | SO-ILE  (N=11) | MO-ILE  (N=11) | p-value | % Change over study weeks | | | | | | | | |
| --- | --- | --- | --- | --- | --- | --- | --- | --- | --- | --- | --- | --- |
|  |  |  |  | **Week 4** | | | **Week 8** | | | **Week 12** | | |
|  |  |  |  | **SO-ILE**  **(N=11)** | **MO-ILE**  **(N=11)** | **p-value** | **SO-ILE**  **(N=11)** | **MOL-ILE**  **(N=11)** | **p-value** | **SO-ILE**  **(N=9)** | **MO-ILE**  **(N=9)** | **p-value** |
| PN days/Wk, days | 7±0 | 6.8±0.3 | 0.103 | 0 | 0 | - | 0 (0,0) | 0 (0,0) | 0.166 | 0 (0,0) | 0 (0,0) | 0.342 |
| Total volume, ml | 2000.7±721.4 | 1945.8±450.9 | 0.832 | 0 (0,12.5) | 0 (0,0) | 0.599 | 0 (0,22.7) | 0 (0,0) | 0.143 | 0 (0,15) | 0 (0,0) | 0.181 |
| Amino acids, g/d | 78.4±19.3 | 87.2±19.2 | 0.3 | 0 (0,0) | 0 (0,0) | 0.363 | 0 (0,0) | 0 (0,0) | 0.406 | 0 (0,10.3) | 0 (0,0) | 0.390 |
| Calorie, kcal/d | 395.6±316 | 348.6±77.1 | 0.641 | 0 (0,0) | 0 (0,0) | 0.363 | 0 (0,9) | 0 (0,0) | 0.216 | 0 (0,10.3) | 0 (0,0) | 0.390 |
| Amino acid, g/kg/d | 1.44±0.25 | 1.45±0.28 | 0.936 | 0 (0,0) | 0 (0,0) | 0.098 | 0 (0,4.1) | 0 (0,2) | 0.942 | 0 (-0.4,9.4) | 0 (0,9.8) | 0.929 |
| Dextrose, g/d | 240.3±28.4 | 231.7±42.0 | 0.582 | 0 (0,0) | 0 (0,0) | 0.188 | 0 (0,0) | 0 (0,0) | 0.654 | 0 (0,19.3) | 0 (0,0) | 0.562 |
| Calories (dextrose), kcal | 816.8±96.5 | 787.5±142.9 | 0.579 | 0 (0,0) | 0 (0,0) | 0.188 | 0 (0,0) | 0 (0,0) | 0.654 | 0 (0,19.3) | 0 (0,0) | 0.562 |
| Fat emulsion, g | 42.6±6.9 | 45.4±8.1 | 0.391 | 0 (0,0) | 0 (0,0) | 0.188 | 0 (0,0) | 0 (0,0) | 0.720 | 0 (0,3.5) | 0 (0,0) | 0.954 |
| Calories (ILE), kcal | 426.1±69.9 | 453.8±82.4 | 0.406 | 0 (0,0) | 0 (0,0) | 0.188 | 0 (0,0) | 0 (0,0) | 0.720 | 0 (0,3.5) | 0 (0,0) | 0.954 |
| Lipids, days/Wk | 6.8±0.3 | 6.3±1.0 | 0.139 | 0 (0,0) | 0 (0,0) | 0.363 | 0 (0, -28.5) | 0 (0,0) | 0.530 | 0 (0,0) | 0 (-39.2,0) | 0.207 |
| Wk PN calorie, kcal/d | 1544.4±181.7 | 1532.2±237.3 | 0.893 | 0 (0,0) | 0 (0,0) | 0.583 | 0 (-7.6,0) | 0 (-13.5,0) | 0.432 | 0 (-2.3,9.7) | 0 (-11.8,0) | 0.158 |
| Wk PN calorie, kcal/kg/d | 29.6±7.8 | 25.3±8.1 | 0.221 | 0 (0,0) | 0 (0,0) | 0.329 | 0 (-9.5,5.4) | 0 (-10,1.9) | 0.688 | 0 (-10.6,10.5) | 0 (-9.6,0.9) | 0.645 |
